# Fecal Microbiota Transplantation for Ulcerative Colitis: A Systematic Review and Meta-Analysis

**DOI:** 10.1371/journal.pone.0157259

**Published:** 2016-06-13

**Authors:** Yanqiang Shi, Yiwei Dong, Wenhui Huang, Decong Zhu, Hua Mao, Peizhu Su

**Affiliations:** 1 The Second Clinical Medical School, Southern Medical University, 510280, Guangzhou City, Guangdong Province, China; 2 Department of Gastroenterology, Zhujiang Hospital, Southern Medical University, 510280, Guangzhou City, Guangdong Province, China; University of South Carolina School of Medicine, UNITED STATES

## Abstract

**Background:**

Fecal microbiota transplantation (FMT) has been recognized as a novel treatment for ulcerative colitis (UC). However, its efficacy and safety remain unclear.

**Objective:**

We conducted this systematic review to assess the efficacy and safety of FMT in UC.

**Data Sources:**

PubMed, EMBASE, Cochrane Central, Web of Science Core Collection, and three other Chinese databases were searched for reports of FMT in UC with clear outcomes.

**Data Extraction and Synthesis:**

We estimated pooled rates [with 95% confidence interval (CI)] of clinical remission among 15 cohort studies and clinical response among 16 cohort studies.

**Results:**

Twenty five studies (2 randomized controlled trials, 15 cohort studies, and 8 case studies) with 234 UC patients were included. Overall, 41.58% (84/202) patients achieved clinical remission (CR) and 65.28% (126/193) achieved clinical response. Among the cohort studies, the pooled estimate of patients who achieved CR and clinical response were 40.5% (95% CI 24.7%-58.7%), and 66.1% (95% CI 43.7%-83.0%). Most adverse events were slight and self-resolving. The analyses of gut microbiota in 7 studies showed that FMT could increase microbiota diversity and richness, similarity, and certain change of bacterial composition.

**Conclusion:**

FMT provides a promising effect for UC with few adverse events. Successful FMT may be associated with an increase in microbiota diversity and richness, similarity, and certain change of bacterial composition.

## Introduction

Ulcerative colitis (UC) is a chronic, relapsing and remitting disease characterized by the overaggressive inflammatory response contributing to the destruction of the gastrointestinal tract. Its main symptoms include bloody diarrhea, abdominal pain, urgent and tenesmus,[1–3] which produce a miserable influence on the quality of life.

While the exact etiology of UC remains unclear, patients with UC are found to have decreased microbiota diversity and species richness leading to the unbalance between adaptation to environment changes and resistance to natural disturbances. [4] The dysbiosis of UC is also characterized by some alterations of bacterial composition, including decrease in *Bacteroidetes*, along with *Firmicutes* (in particular *Clostridium IXa and IV groups*, *Bifidobacteria*, *Lactobacillus* and *Faecalibacterium prausnitzii*), and an increase in *Proteobacteria* and *Actinobacteria*.[5–7]

The human gut contains, in the assortment of 1000 bacterial species, 100-fold more genes than the human genome. [8] Multifaceted microbial population is considered as an organ with critical function in human health. It has been demonstrated that the changes in gut microbiota reduce the ability of the intestinal environment to fight pathogens and can be relevant with some disease conditions. [9–11]

With the deep research into the interaction of gut microbiota and host, probiotics, prebiotics and symbiotics began to be used in UC patients to promote intestinal microbiota homeostasis. [12] Some randomized controlled trials (RCTs) and cohort studies of *Lactobacillus* or VSL#3 have already showed their effect on maintaining UC remission and preventing recurrence. [13–15]This promising therapy contributed to more attention to fecal microbiota transplantation (FMT) because they both belong to bacteria-driven therapy.

FMT, which traced back to the 4^th^ century in China, [16] was first reported formally by Eiseman *et al*. [17] in 1958 for four patients with pseudomembranous colitis. The fecal retention enemas were successful in all patients, which did not respond to antibiotics. During the past decades, FMT has been applied for refractory Clostridium difficile infection (CDI) on the basic of the idea that the normal microbiota community can be rebuilt by importing the colonic microbiota of the healthy person. Among numerous trials, FMT was reported to be more effective than vancomycin for recurrent CDI patients and its mean successful rate was 87% to 90% for the >500 cases reported. [18–21] IBD patients are at a higher risk for the development of CDI-associated diseases; therefore, FMT has been regarded as a potential treatment for IBD. Several retrospective trials of FMT in IBD showed attractive results, especially for patients with UC.

The majority of trials about FMT for UC or IBD are one-armed cohort studies or case series. The first two RCTs evaluating the efficacy and safety of FMT were recently published on the *Gastroenterology* in 2015. [22, 23] In the most recent systematic review, Colman *et al*. [24] found a clinical remission rate of 36.2% in IBD patients for FMT only in cohort studies. Subgroup analysis demonstrated a pooled estimate of clinical remission of 22% for UC. Additionally, several relevant studies were reported after the last systematic review in 2014. As a form of IBD, UC has its own genetic, pathogenic characteristic resulting in its own therapeutic identity. Given that there has been no systematic review focusing only on UC subjects, we performed a systematic review with the most up-to-date and reliable evidence to evaluate the efficacy and safety of FMT only for patients with UC.

## Materials and Methods

This systematic review adheres to the Preferred Reporting Items for Systematic Reviews and Meta-Analyses statement (PRISMA) and Cochrane Handbook (Version 5.0.2) (S1 File). [25] Methods of the analyses and inclusion criteria were specified in advance and documented in a protocol. This protocol was registered at PROSPERO (CRD42015025076) (S2 File).

### Eligibility criteria

The following inclusion criteria were used: (I) patients of any age with ulcerative colitis undertaken FMT; (II) studies comparing FMT with placebo, standard care or without a control group; (III) studies that clearly described endpoints; (IV) journal articles, letters to the editor, abstracts and proceedings. The exclusion criteria were: (I) animal or in vitro studies; (II) language other than English or Chinese; (III) only included patients suffered from UC with CDI; (IV) data provided for UC patients was not reported separately or the data was overlapped across several studies; (V) interviews and reviews.

### Search strategy

The systematic search was performed in: PubMed, EMBASE, Cochrane Central, Web of Science Core Collection, Chinese Biological Medicine (CBM), China National Knowledge Infrastructure (CNKI) and Wanfang Med Online. The last three databases are in Chinese. All databases were searched up to August, 2015. The following terms were used: “fecal”, “faecal”, “feces”, “faeces”, “microbiota”,” “microflora”, “stool”, “fecal flora”,“faecal flora”, “transplant”, “transplants”, “transplantation”, “transfusion”, “implant”, “implantation”, “instillation”, “donor”, “therapy”,“bacteriotherapy”, “ulcerative colitis”, “UC”, “inflammatory bowel diseases”, “IBD”. Some of the above words were identified by Anderson *et al*. [26] In addition, we also searched *clinical trial*.*gov*. The hand searching of references in relevant reviews was also performed. No limitations were placed on language, study type, publication date and publication status.

### Data Collection

Data was extracted from eligible articles by two authors independently. The following information was extracted in all included articles: (1) characteristics of participants (including total number, age, sex, diagnosis, duration and severity of UC); (2) type of intervention (including number of exposed and unexposed groups, fecal processing, frequency, route of administration, donor relationship and length of follow-up); (3) clinical outcomes (including clinical remission, clinical response, adverse events, mucosal healing and quality of life assessment); (4) alteration of gastrointestinal microbiota in participants (including similarity, diversity and richness, and composition to the donor).

### Methodology quality appraisal

Quality assessment of each RCT and cohort study was carried out by two authors independently and disagreements were resolved by discussion. The methodological quality of two RCTs was assessed using the Cochrane risk of bias tool.[27] For the one-arm cohort studies, quality was assessed by the adjusted Newcastle-Ottawa Scale on the following criteria: representativeness of the UC cohort, ascertainment of FMT, demonstration that outcome of interest was not present at start of study, assessment of outcome, the length (at least 3months) and adequacy of follow-up. [28, 29] It should be noted that we considered “demonstration that outcome of interest was not present at start of study” as “evidence of no prior FMT exposure”.

### Statistical methods

The meta-analysis of the two included RCTs cannot be performed due to their different control groups. Therefore, for RCTs as well as case studies, descriptive summaries of efficacy and safety of FMT were reported. To provide more information for further studies and make comparison with results of RCTs, meta-analysis of cohorts based on random effects model was conducted to evaluate clinical remission rate and clinical response rate with their corresponding 95% confidence interval (CI). The random effects model was conducted using Der Simonian and Laird method.[30] The presence of heterogeneity was assessed using Q statistic (Chi-square test) and the I^2^ statistic was used to assess the degree of heterogeneity between the trials. Funnel plot was employed to measure the potential publication bias. Sensitivity analyses were undertaken in clinical remission and response. Subgroup analysis was stratified by donor type, the route of delivery, and number of treatment. All meta-analyses were performed using Meta-Analyst software (version Beta 3.13; Tufts Medical Center, Boston, MA) to pool effect sizes of the above two main outcomes. [31]

## Results

**Fig 1:**
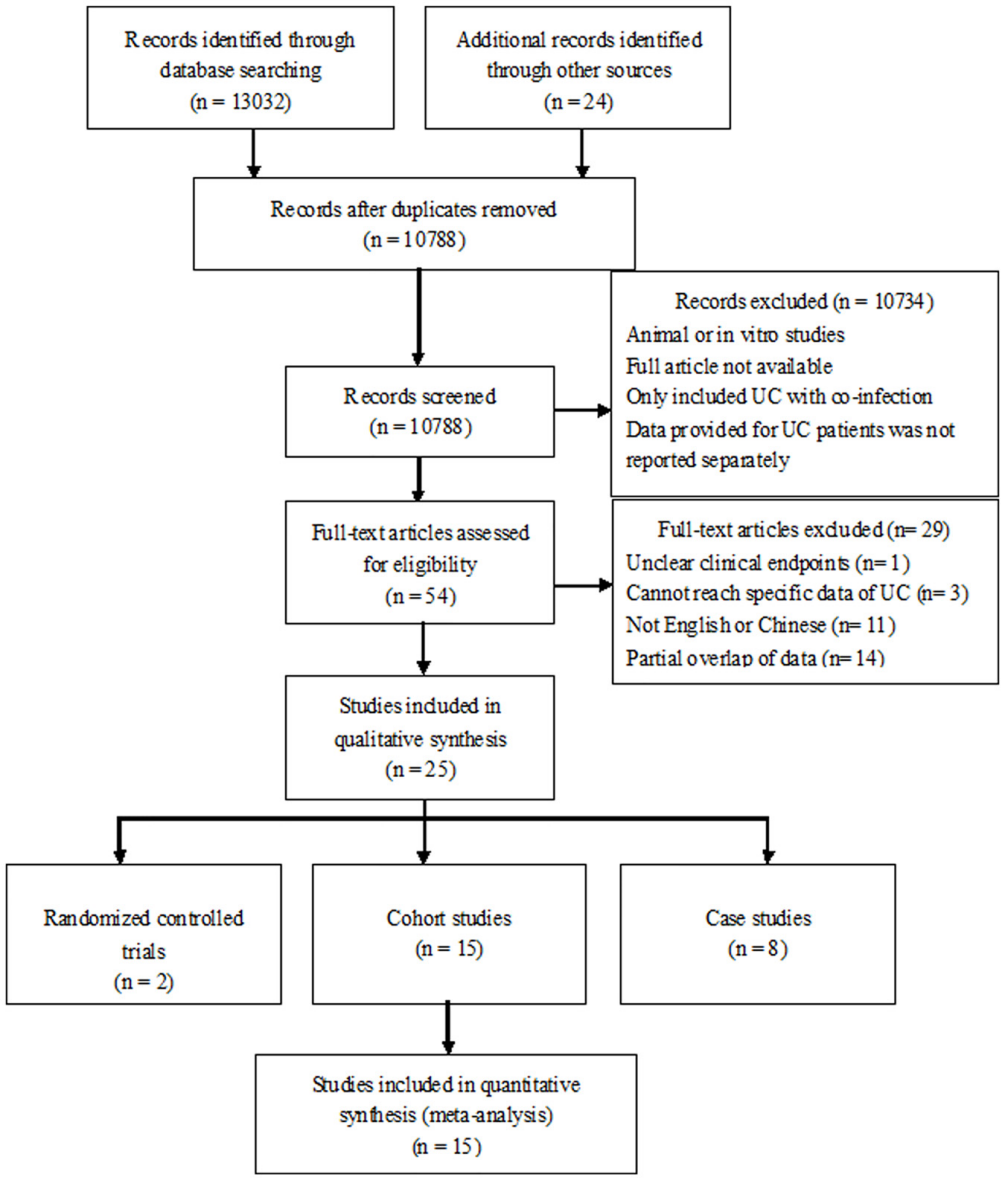
Flow chart of studies of fecal microbiota transplantation in ulcerative colitis.

After duplicate removal, the search identified 10788 records (Fig 1). Based on the screening of titles and abstracts, 54 studies were obtained and reviewed for potential eligibility. Of those, 29 articles were excluded due to their unclear clinical endpoints, nonspecific data of UC, overlap of data or language restriction. Consequently, 25 studies fulfilled the selection criteria, including 2 RCTs, [22, 23] 15 cohort studies [32–46] and 8 case studies [47–54]. Only 6 cohort studies and 3 case studies were abstracts, the other 16 studies were journal articles. Characteristics of each original study were presented in Tables 1, 2, 3 and 4 and S1 Table.

**Table 1:** Characteristic of cohort studies.

t			Characteristics of patients		
Study (Year, Reference)	Country	Type	Number (M/F)	Age	Severity (duration)
Wang *et al*.^39^(2014)	China	Journal article	2	NR	Steroid-dependent or refractory
Kump *et al*.^35^(2013)	Austria	Journal article	6(3/3)	36.5 (17–52)	Therapy-refractory (5.5y)
Kunde *et al*.^36^(2013)	America	Journal article	9(5/4) †	14.8 (7–20)	Mild-moderate (3.8y)
Suskind *et al*.^43^(2015)	America	Journal article	4(4/0)	14.5 (13–16)	Mild-moderate (1y)
Wei *et al*.^44^(2015)	China	Journal article	11(3/8) ‡	47 (26–70)	Mild-moderate (5y)
Karolewska-Bochenek *et al*.^46^(2015)	Poland	Abstract	4 (1/3)	15 (10–17)	Moderate-severe and refractory
Kellermayer *et al*.^41^(2015)	America	Letter	3 (2/1)	15 (14–16)	Immunotherapy-dependent
Angelberger *et al*.^34^(2013)	Austria	Journal article	5 (3/2)	34.2 (22–51)	Moderate-severe (4.1y)
Scaldaferri *et al*.^32^(2015)	Italy	Abstract	8	NR	Mild to moderate
Ren *et al*.^42^(2015)	China	Journal article	7 (6/1)	36.6 (17–66)	Active (9.1y)
Cui *et al*.^45^(2015)	China	Journal article	14 (10/4)	31.6(11–48)	Steroid-dependent
Damman *et al*.^40^(2015)	America	Journal article	6 (2/4)	41.7 (25–61)	Mild-moderate (18.2y)
Borody *et al*.^33^(2012)	Australia	Abstract	62 (40/22)	M 42.3±11.5y; F 48.45±16.49	Active
Kump *et al*.^38^(2013)	Austria	Abstract	9	NR	Active
Landy *et al*.^37^(2013)	United Kingdom	Abstract	8	NR	Refractory pouchitis

NR, not reported.

^†^ One patient was excluded in all extracted data due to enema intolerance.

^‡^ One patient was excluded in all extracted data except age and due to enema intolerance.

**Table 2:** Characteristic of cohort studies. (Continued)

	Characteristics of intervention				
Study (Year, Reference)	Patient preparation	Donor	Stool processing	The route of delivery	Number of treatment
Wang *et al*.^39^(2014)	Proton pump inhibitor	Nominated by pts(family members/close friends)	Total 150 mL infused	Gastroscopy	Every other day ×3
Kump *et al*.^35^(2013)	Lavage with standard PEG solution	Nonrelatives, different household	100-150g/200-500mL saline/total 300-500mL infused	Colonoscopy	×1
Kunde *et al*.^36^(2013)	None	Family member or close friend	90g (70–113 g) / 250mL saline/total 240mL infused	Enema(60ml/15 minutes, 1 hour)	Daily ×5
Suskind *et al*.^43^(2015)	Rifaximin, omeprazole and MiraLAX	NR	30g/100mL saline / 30mL infused	Nasogastric tube	×1
Wei *et al*.^44^(2015)	Vancomycin/PEG	Nonrelatives, different household	60g/350mL saline/total 300mL infused	Colonoscopy	×1
Karolewska-Bochenek *et al*.^46^(2015)	Proton pump inhibitor	Nonrelatives	Total 50mL infused	Gastroscopy	8 infusions in 14 days
Kellermayer *et al*.^41^(2015)	NR	Standardized single donor	50g/250mL saline	Colonoscopy + enemas	22–30 treatments (tapering course)
Angelberger *et al*.^34^(2013)	Metronidazole 5–10 days; pantoprazole	Nominated by pts(no family/health-care staff)	NJ: 24g (17-25g) /250mL; enema:20g (6-22g)/100mL	NJ + enema	×3 consecutive days
Scaldaferri *et al*.^32^(2015)	NR	Nominated by pts	Fecal slurry (200cc)	Colonoscopy	×3
Ren *et al*.^42^(2015)	NR	Relatives/healthy volunteers	Gastroscopy: 200-300mL infused; colonoscopy: 100-200Ml infused	Gastroscopy + colonoscopy	5 pts×1; 1 pt×2; 1 pt×3
Cui *et al*.^45^(2015)	Metoclopramide; esomeprazole magnesium	Relatives or friends	500–1000 mL saline/150-200 mL suspension infused	Gastroscopy	10 pts×1; 4 pts×2
Damman *et al*.^40^(2015)	Without antibiotic pretreatment; after a GoLYTELY bowel purge.	Family member or close friend	2-3mL saline per gram of stool/total 175 to 290 cc infused	Colonoscopy	×1
Borody *et al*.^33^(2012)	NR	NR	NR	NR	NR
Kump *et al*.^38^(2013)	Antibiotic triple therapy for 10 days	Healthy volunteer	NR	Colonoscopy +sigmoidoscopy	×5
Landy *et al*.^37^ (2013)	NR	Nominated by pts	30g/50mL saline	Nasogastric tube	×1

NR, not reported; NJ, nasojejunal tube.

**Table 3:** Characteristic of cohort studies. (Continued)

	Characteristics of outcomes		
Study (Year, Reference)	Clinical outcome	Adverse	Follow-up
Wang *et al*.^39^(2014)	Clinical remission (1/2 1m, 3m); Clinical response (2/2 1w).	None	3 months
Kump *et al*.^35^(2013)	Clinical remission (0/6 90d); Clinical improvement (6/6 2w, 4/6 stool frequency increased 30d, 2/6 sustained improvement 90d); Total colectomy (1/6) and total proctocolectomy (2/6).	Increased stool frequency and self-limiting fever (1/6)	90 days
Kunde *et al*.^36^(2013)	Clinical remission (3/9 1w and 4w); Clinical response (7/9 1w, 6/9 1m)	Bloating/ flatilence (9/9), abdominal pain/cramping (6/9), diarrhea (6/9), blood in stool (3/9), fatigue (3/9), fever (2/9).	4 weeks
Suskind *et al*.^43^(2015)	None clinically improved with FMT; With additional standard medical therapies, clinical remission (0/4 2w, 1/4 6w, 2/4 12w)	Nasal stuffiness (1/4), bloating (1/4), flatulence (1/4), vomiting (2/4), C *difficile* diarrhea (2/4).	12 weeks
Wei *et al*.^44^(2015)	Mean Mayo score decreased from 5.80±1.87 to 1.50±1.35 (*P* < 0.01)	Self-limiting fever (2/10).	4 weeks
Karolewska-Bochenek *et al*.^46^(2015)	All patients achieved clinical improvement (PUCAI), but none achieved complete remission.	Vomiting (3/4)	4 weeks
Kellermayer *et al*.^41^(2015)	All patients obtained remission for more than 11 weeks, but finally experienced a relapse acquiring immunotherapy.	Bloody stools and cramping (1/3)	120-220days
Angelberger *et al*.^34^(2013)	Clinical remission(0/5 12w); Clinical response (1/5 12w); further deterioration(2/5 4w)	Fever (5/5), sore throat (5/5), flatulence (2/5), vomiting (1/5), common cold (3/5), pancreatitis (1/5), itchiness (1/5), erythema (1/5), paresthesia of the hip (1/5), collapse due to orthostatic disorder (1/5), blisters on the tongue (1/5).	12 weeks
Scaldaferri *et al*.^32^(2015)	Clinical remission(2/8 2w; 2/8 6w; 3/8 12w); Clinical response(2/8 2w;4/8 6w;4/8 12w); Endoscope response(2/6)	Kidney stone (1/6), disease worsening (2/8)	12 weeks
Ren *et al*.^42^(2015)	A11 patients achieved reduction of Mayo scores 7, 4, 6, 5, 6, 9 and 9 respectively	Fever (3/7), flatulence (4/7), diarrhea (2/7), monilia albicans and proteus mirabilis infection (1/7).	30–210 days
Cui *et al*.^45^(2015)	Clinical improvement and being free-steroid (8/14); long-term remission (4/14).	Fever (2/14), diarrhea(2/14), testicular pain(1/14)	3–18 months
Damman *et al*.^40^(2015)	Clinical remission (1/6 4w; 0/6 3m); Worsening symptoms (6/6 3m); Histology scores improvement (5/6 4w)	Abdominal cramping and stool frequency (several patients); Micro-perforation (n = 1 disenrolled)	3 months
Borody *et al*.^33^(2012)	Complete clinical remission (42/62); Partial response (15/62); Failure (5/62); Normalization of mucosa (8/21).	No significant adverse events.	NR
Kump *et al*.^38^(2013)	Reduction of the Mayo score >3 points (5/9 90d); Sustained mucosal healing (1/9); Failed a sustained clinical improvement (4/9).	None of the patients suffered any severe adverse events.	90 days
Landy *et al*.^37^ (2013)	Clinical remission (0/8); Improvement in CGQoL (0/8)	NR	4 weeks

NR, not reported; FMT, fecal microbiota transplantation; CGQoL, cleveland golbal quality of life score.

**Table 4:** Characteristics of randomized controlled trails.

Characteristic	Moayyedi *et al*.^22^	Rossen *et al*.^23^
Study design	Double-blind randomized controlled (1:1)	Double-blind randomized controlled (1:1)
Patients		
Number and Age	FMT: 38/42.2±15 Placebo(water): 37/35.8±12.1	FMT-don: 23/40.0 FMT-aut: 25/41.0
Severity	Adult patients with active mild-moderate UC	Adult patients with mild-moderate UC
Concomitant medications	Mesalamine, glucocorticoids, immunosuppressive and anti-TNF (at a stable dose ≥12weeks)	Anti-TNF and methotrexate within 8 weeks before inclusion and cyclosporine within 4 weeks before inclusion were not allowed
Intervention		
Patient preparation	NR	Bowel lavage (2L macrogol solution and 2L clear fluids)
Donor	One patient's spouse, the others were 6 volunteers	Healthy partners, relatives or volunteers
Stool processing	50g/mixed with 300ml water/infusion 50ml	120g/500ml saline
Delivery and Frequency	Retention enema/weekly ×6	Nasoduodenal tube/2 treatments (at 1 day and 3 weeks later)
Clinical outcome		
Outcome definition	Clinical remission: full Mayo score <3 points and an endoscopic Mayo score = 0 at week 7; Clinical response: a reduction in full Mayo score ≥3 points	Primary end point: clinical remission and Mayo endoscopic score of the sigmoid and rectum improved ≥1 point at 12 weeks; Secondary end points: clinical remission (SCCAI ≤2 points) and clinical response (a reduction of SCCAI ≥1.5 points) at 6 and 12 weeks.
Clinical remission	FMT: 9/38(24%) vs Placebo: 2/37(5%) (*P* = .03)	FMT-don: 7/23(30.4%) vs FMT-aut: 8/25(32.0%)
Clinical response	FMT: 15/38(39%) vs Placebo: 9/37(24%) (*P* = .16)	FMT-don: 11/23(47.8%) vs FMT-aut: 13/25(52.0%)
Other outcomes	IBDQ score: FMT(149.38) vs Placebo(152.13) (*P* = .44); EQ-5D score: FMT(70.07) vs Placebo(68.52) (*P* = .99)	Primary end point: FMT-don 7/23(30.4%) vs FMT-aut 5/25(20.0%) (*P* = .51); Endoscopic outcome: FMT-don 8/23(34.7%) vs FMT-aut 9/25(36.0%) (*P* = 1.0)
Adverse events	Significant adverse events (n = 5): worsening colitis and urgent colectomy (Placebo: n = 1); patchy inflammation of the colon and rectal abscess formation (FMT: n = 2, Placebo, n = 1); worsening abdominal discomfort and C difficile toxin (+) (FMT: n = 1).	Discomfort tube placement (1 vs. 1); Fever (2 vs. 0); Nausea (2 vs. 1), Malaise (0 vs. 1); Increase of stool frequency/diarrhea (5 vs. 1); Headache (1 vs. 1); Vomited fecal infusion (2 vs. 0); Vomited bowel preparation (1 vs. 0); Vomiting (1 vs. 0); Abdominal cramps (0 vs. 6); Abdominal pain (1 vs. 4); Abdominal murmurs (4 vs. 8); Dizziness (0 vs. 1); Mild constipation(1 vs. 0). Serious adverse events (n = 4): perforation, small bowel Crohn's disease, primo cytomegalovirus infection and cervix carcinoma.

FMT, fecal microbiota transplantation; UC, ulcerative colitis; NR, not reported; IBDQ, Inflammatory Bowel Disease Questionnaire score; EQ-5D, EuroQol score. SCCI, Simple Clinical Colitis Activity Index.

### Methodological quality of included studies

The methodological quality of two RCTs was showed in S2 Table. The RCT conducted by Rossen et al. was rated as low risk of bias on five out of seven items, and the study of Moayyedi *et al*. on three items. The total scores of each cohort study ranged from 3 to 5 points with a mean score as 4.3. (S2 Table), indicating the quality of single-arm studies was medium to high. All the cohort studies showed ascertainment of exposure and assessment of outcome. 1 study did not have adequacy of follow-up and 5 studies did not report enough length of follow-up as long as three months. Seven of 15 studies were considered to have representativeness of the exposed cohort. Only 3 studies demonstrated that outcome of interest was not present at start of study.

### Patient demographics

Among the 25 studies, of the 234 patients treated with FMT, 3 patients were excluded due to enema intolerance (n = 2 patients) or micro-perforation (n = 1 patient). [36, 40, 44] The age range of the remaining 231 patients varied widely from 18 months to 66 years. As for the severity, 98(42.4%) patients were described as “mild or mild-moderate disease”, 47(20.3%) as “moderate-severe or severe disease”. Other descriptions of the disease severity included 149(64.5%) “active disease”, 19(8.2%) “therapy dependent” and 26(11.3%) “therapy refractory”. And the duration of UC extended from 1 year to 40 years. Clinical follow-up time of participants ranged from 3 weeks to 13 years.

### Patient preparation

Patient preparations were reported in 14 of 25 studies (n = 100 patients) and were unclear in the other 11 studies (n = 131 patients). 6 studies (n = 35 patients) received antibiotic pretreatment before FMT, such as vancomycin, rifampicin and omeprazole, while 2 studies including 15 patients declared that any antibiotic pretreatment was not allowed. [36, 40] As for bowel lavage, polyethylene glycol was used in 52 patients of 5 studies. [23, 35, 40, 44, 49] Other than the above two kinds, proton pump inhibition therapy was used to inhibit the secretion of gastric acid in 4 studies (n = 25 patients) as another kind of patient preparations before FMT procedure via gastroscopy or nasojejunal tube. [34, 39, 45, 46]

### Fecal microbiota transplantation characteristics

Donor identification was reported in 20 studies (n = 162 patients), including related donor (n = 55 patients), unrelated donor (n = 75 patients) and unclear relationship (n = 32 patients). Related donors are traditionally dominated by patients including family member, partner, spouse and close friend. And health screening for donors was carried out in 18 studies (n = 155 patients). The information of stool processing was available in 17 studies, including the amount of fecal infused into normal saline (9.6-100g/100mL) and the volume of fecal suspension in per treatment (30 to 500mL).

Routes of administration included enema in 5 studies, colonoscopy in 4 studies, gastroscopy in 3 studies, nasogastric tube in 2 study, nasoduodenal tube in 1 study, percutaneous endoscopic cecostomy in 1 study and combination of two routes in 5 studies. The number of total FMT treatments in each patient was categorized into three levels: only 1 treatment (n = 7 studies), more than 1 treatment (n = 15 studies) and unclear times (n = 3 studies). In the RCT of Moayyedi *et al*., [22] FMT was performed via enema weekly for 6 weeks with significant outcome for participants. In the RCT of Rossen *et al*., [23] FMT was performed via nasoduodenal tube and there was no significant statistically difference at 0 and 3 weeks.

### Therapeutic effect

Overall, data from all included studies showed that the percentages of clinical remission and clinical response were 41.58% (84/202), 65.28% (126/193) respectively.

In the RCT performed by Moayyedi *et al*., [22] FMT (9/38, 24%) achieved significantly better clinical remission than those receiving water (2/37, 5%) at week 7 (P = 0.03). In RCT conducted by Rossen *et al*., [23] 30.4% patients (7/23) receiving FMT from healthy donors (FMT-D group) and 20.0% patients (5/25) receiving FMT from their own fecal microbiota (FMT-A group) achieved clinical remission and endoscopic response (P = .51). 47.8% patients (11/23) in the FMT-D group and 52.0% patients (13/25) in the FMT-A group had a clinical response. Besides, there was no statistical significance between the two groups on clinical response, clinical remission or endoscopic outcomes.

### Meta-analysis of cohort studies

#### Clinical remission and clinical response

**Fig 2:**
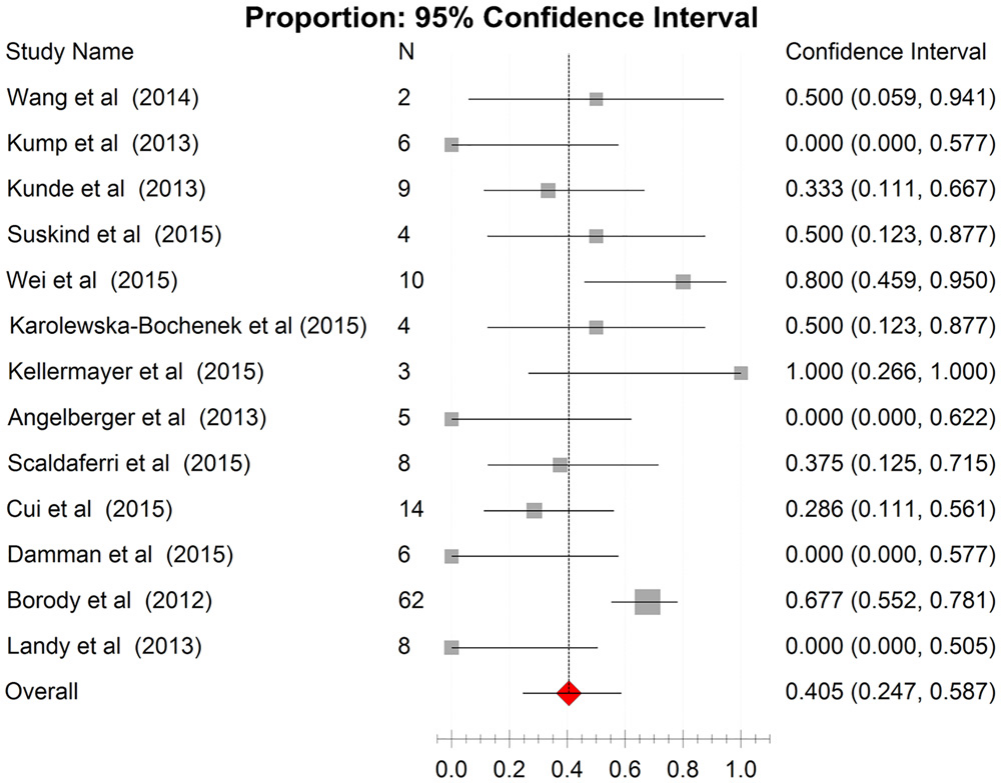
Forest plot of all cohort studies in clinical remission. Pooled estimate of 40.5% (95% CI 24.7%-60.4%).

**Fig 3:**
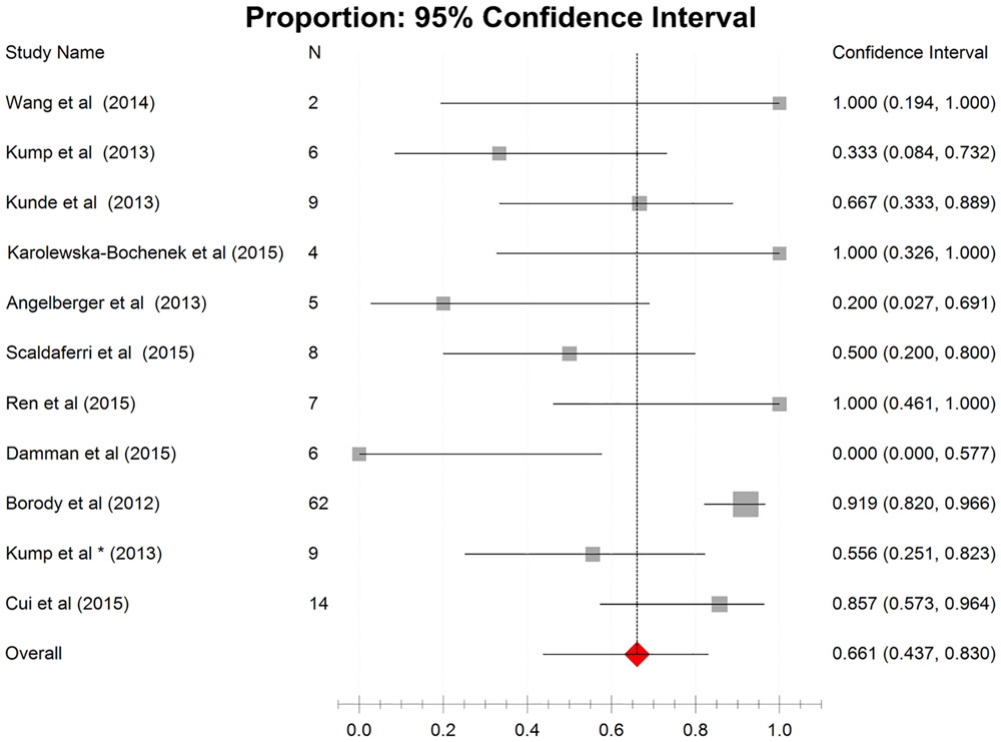
Forest plot of all cohort studies in clinical response. Pooled estimate of 66.1% (95% CI 43.7%-83.0%).

The results of the synthesis of outcomes from cohorts were shown in Fig 2 and in Fig 3. Data on clinical remission and clinical response were extracted from 13 studies assessing 141 patients and 11 studies assessing 132 patients respectively. The pooled estimate of clinical remission rate was 40.5% (95% CI: 24.7%-58.7%) with low heterogeneity (Cochrane’s Q, P = 0.005; I^2^ = 36.5%), whereas the pooled estimate of clinical response rate was 66.1% (95% CI 43.7%-83.0%) with moderate heterogeneity (Cochrane’s Q, P = 0.001; I^2^ = 40.2%).

#### Sensitivity analyses and assessment of bias

In the meta-analysis of clinical remission and clinical response, the result of leave-one-out analysis did not materially change the overall effect, indicating that clinical remission rate and clinical response rate were not sensitive to any individual study included in the analysis. This result showed the robustness of our evidence. The funnel plots showed evidence of asymmetry revealing possible existence of publication bias. (S3 File)

#### Subgroup analysis

Subgroup analyses were conducted for donor type, the route of delivery, and number of treatment. The details of all subgroup analyses were shown in S3 File. Differences in these subgroups were not associated with heterogeneity in the estimated clinical remission rate and clinical response rate.

#### Mucosal healing

Mucosal healing was assessed in terms of endoscopy and histology. The descriptions of mucosal healing were “mucosal healing”, “endoscopic remission” (Mayo endoscopic subgroup sore = 0) and “normal”, while the description of histology just included not active inflammation. Overall, the mucosal healing was reported in 25 of 81 (30.87%) patients in endoscopy and 30 of 48 (62.50%) patients in histology. In one RCT, 7 patients in remission had no active inflammation and the remaining 2 patients in remission had mild patchy inflammation. [22]

#### Quality of life assessment

Of the 25 studies included, 4 studies including 2 cohort studies and 2 RCTs reported quality of life assessment. [22, 23, 37, 44] In the cohort study conducted by Wei *et al*., [44] the mean Inflammatory Bowel Disease Questionnaire score (IBDQ) score increased significantly to 177.30±20.88 at 4 weeks after FMT. With regard to Landy *et al*, [37] there was no improvement in Cleveland Global Quality of Life Score (CGQoL). In the RCT conducted by Moayyedi *et al*., [22] the IBDQ and EuroQol score (EQ-5D) were not statistically significant between FMT group and placebo group at 6 weeks. In the RCT performed by Rossen *et al*., [23] the total IBDQ score improved shortly in responders after treatments with statistical significance but no change was found in nonresponders.

### Safety and adverse events

Adverse events were monitored and reported in 19 of 25 studies. Among 19 studies, no severe adverse event was noted in 3 studies after FMT without more details.[33, 38, 53] On the whole, FMT was of safety and tolerance. Fever occurred in 18 patients from 8 studies after FMT. Fever severity was mild to moderate or self-resolving in most patients, while 2 subjects from 2 studies required antibiotics or antivirus to deal with the fever caused by infection. [42, 52] Patients in 2 studies suffered from nasal stuffiness and sore throat probably due to nasogastric tube or nasojejunal tube placement. [34, 43] Additionally, C *difficile* diarrhea was reported in 2 patients from the cohort study conducted by Suskind *et al*. [43] The majority of adverse events were self-resolving including bloating, abdominal pain, cramping, blood in stool, diarrhea and fatigue.

In the RCT conducted by Moayyedi *et al*., [22] 3 patients treated with FMT developed adverse events including colonic patchy inflammation (n = 2 patients), rectal abscess (n = 2 patients) and worsening abdominal discomfort (n = 1 patient). In the RCT performed by Rossen *et al*. with 78.3% adverse events related to FMT, most events were self-resolving within 2 days including fever. [23] Additionally, 2 patients in the intervention group developed serious adverse events including severe small bowel Crohn’s disease and cervix carcinoma, but these were not related to the FMT. [23]

### Microbiota analysis

Effects on gastrointestinal microbiota in UC after FMT were reported in 7 studies including 2 RCTs and 5 cohort studies. [22, 23, 34, 35, 40, 41, 45] Similarity to donors was reported in 16 of total 21 patients in 4 cohort studies in the condition of positive response to FMT, except one trial conducted by Kump *et al*. showed no clinical improvement in 3 of 7 patients. [34, 35, 40, 45] Moayyedi *et al*. demonstrated that patients in the intervention group received higher similarity to their related donors than the control group. [22] Data from Rossen *et al*. indicated that responders in FMT-D group had a significant higher similarity to their donors than non-responders. [23] Among the 16 patients, 10 patients were reported to experience a long-term resemblance more than 90 days; 5 patients experienced a rapid slight or moderate increase with subsequent decline, even back to the baseline at 2–3 months.

5 studies reported the change of microbiota diversity and richness, using the Shannon diversity index or the number of different operational taxonomic units (OTUs). [23, 35, 40, 41, 45] 4 trials, which had specific data for every subject respectively, demonstrated the increase diversity and richness in 17 of total 36 patients after FMT from healthy donors. [23, 35, 41, 45] Studies from Damman *et al*. showed that there was no significant difference in diversity among subjects after transplantation compared to the baseline. [40] In addition, Kump *et al*. found that the change was temporal, meaning that the diversity as well as richness peaked at day 7 and thereafter back to baseline. [35]

The alteration of microbiota composition after positive FMT treatment has been the central point in recent trials and 6 studies we included focused on it. [23, 34, 35, 40, 41, 45] The magnitude and persistence of the alterations in baterial composition were highly variable between patients. On the phylum level, the increase of *Bacteroidetes* was observed in 3 trials, [35, 40, 45] while the decrease of *Bacteroidetes* in the responders in FMT-D group in 1 RCT. [23] Evidence also showed that the increase of *Actinobacteria*, *Firmicutes* and *Clostridium clusters IV*, *XIVa*, *XVIII* but the decrease of *Proteobacteria* in some subjects. [23, 35, 40, 45] On the family level, the increase of *Lachnospiraceae* was observed in 2 trials and the increase of *Bacteroidaceae* was reported in Kump *et al*. [34, 35, 41] The reduction of *Enterobacteriaceae* was demonstrated in 2 trials. [34, 35] On the genus level, Kellermayer *et al*. showed the increase of *Coprococcus*. [41] Additionally, Angelberger *et al*. and Kellermayer *et al*. found that, at the family level, the abundance of *Enterobacteriaceae* and *Lachnospiraceae* were associated with clinical outcomes or UC disease activity. [34, 41] In the study of Kump *et al*. the alterations of bacterial composition had no association with clinical performance, [36] while Cui et al showed that the degree of microbiota reconstruction was associated with the clinical response in the patients with steroid-dependent UC. [45]

## Discussion

This systematic review involved 231 patients from 2 RCTs, 15 cohorts and 8 case studies. For clinical remission and response in 2 RCTs, Moayyedi and his colleagues reported a statistically significant effect of 24% and 39% respectively, comparing with the study of Rossen et al., which reported effects of 30.4% and 47.8% respectively with no statistically significant. In comparison with the results of RCTs, meta-analysis based on 15 cohorts with poor quality showed higher clinical remission and response rate of 40.5% and 66.1% respectively. The results from RCTs and cohort studies showed similarly high clinical response and clinical remission in FMT for the treatment of UC, showing that FMT is a promising therapy to some extent. According to microbiota analysis of 2 RCTs and 5 cohorts, FMT generally resulted in a manipulation of microbiota towards donor microbiota, especially in the patients with favorable outcomes. However, these results needed further certification with more well-designed RCTs.

As for heterogeneity in meta-analysis, the I^2^ statistic revealed that heterogeneity was moderate and sensitivity analysis showed the robustness of results. But the subgroup analyses did not find the specific causes of heterogeneity. In short, the results of meta-analysis were trusted and believed to some degree. And the important clinical heterogeneity, including age of patients, the choice of donor, and the route and number of treatments administered, were well stressed and discussed as follows.

### Children and adolescents

Children and adolescents have vulnerable nature and their gut microbiota might not be as stable as adults. These characteristics might be beneficial to implantation and manipulation of gut microbiota in younger people. But children and adolescents may have more risks than adults due to the unknown causal associations between gut microbiota and a number of diseases. The unknown long-term effects on children and adolescents also need to be taken into account. [55]

### Donor selection and screening

Donor selection is one of the crucial unresolved questions in FMT. Related donors share common genetic and/or environmental factors with recipients, so they could have greatest common microbial species with the recipients. This similarity minimized the risk of transmission of infectious diseases and led to more tolerance of FMT. However, there is a possibility that the similar genetics and environments lead to altered microbiota of the related donor, though the donor does not suffer from UC. On this condition, the altered microbiota of related donors may result in re-development of UC in patients; therefore the unrelated donors may be preferable in UC. In addition, unrelated donors reduce screening cost. Therefore unrelated donors rather than related donors facilitate and standardize the process of FMT if industrialization come true one day. [55] Due to the differences between CDI and UC, the screening methods for selection of donors in CDI are not adequate in UC and more aspects should be taken into consideration in UC. Trials with high quality investigating the effect of donor types on outcomes are surely needed.

### The route of delivery

Delivery method could be a key factor affecting the efficacy of FMT. A number of delivery routes have been used in FMT for UC: gastroscopy, nasogastric / nasojejunal tube, colonoscopy and retention enemas. Previous studies and reviewers have suggested a slight superiority of the colonoscopy in CDI patients but without sufficient evidence. [19] Although some hypotheses said the gastric acid of upper delivery may denature microbiota, such as Bacteroidetes and Firmicutes, [56] the exact association between them has not been verified 7 cohort studies and 1 RCT involved reported the upper gastrointestinal delivery, including gastroscopy in 3 studies, nasogastric tube in 2 studies, the combination of nasojejunal tube and enema in 1 study, the combination of gastroscopy and colonoscopy in 1 study and nasojejunal tube in the RCT. Among them, 4 of 7 cohort studies reported the use of proton pump inhibitor to inhibit the secretion of gastric acid before and during the FMT procedure, and the use of nasojejunal tube or gastroscopy rather than nasogastric tube can deliver microbiota directly into the mid-guts. Therefore, we consider the influence of gastric acid to be limited. The concerns for the use of nasogastric / nasojejunal tube were: small volumes, vomiting, aspiration, injury of upper gastrointestinal tract and the necessity to be verified by x-ray before transplant. Colonoscopy could visualize the relevant pathology and deliver larger volume suspensions directly into the site of inflammation in the colon, while endoscopy procedure increases the risk of perforation. In addition, the retention of the infused material via colonoscopy was better than via enema. Enema is accessible, safe and inexpensive, but intolerance was reported in some patients. [21] Additional well-designed trails are needed to identify the best route of delivery.

### Number of treatment

Number of treatment to obtain beneficial outcome in UC also constitutes additional concerns. However, most of cured patients with CDI only received single administration of FMT. [21] UC is a chronic, relapsing and remitting disease; therefore unlike CDI, gut microbiome in UC was stable and resilient to change on condition that the manipulation of gut microbiome was in short-term. This phenomenon suggested that several treatments or more drastic microflora manipulation is needed to permanently reconstitute and remain a balance bacterial community in UC patients. [4] More high quality trials are required to determine the number of treatments needed for different UC patients.

### Microbiota change

FMT generally produced a major alteration in the patients’ microbiota towards donor microbiota, especially in the patients with favorable outcomes. This suggested that donor selection required more attention due to their potential influence on outcomes. The similarity maintained over 2–3 months over half of the patients. However, in some subjects, the increased similarity was transient and not stable for a long time. In addition, the patients who shared the increased similarity had different clinical outcomes, suggesting that the only presence of healthy microbiota signature was not sufficient to lead to positive effects of FMT. Thus, the exact alterations of composition, diversity and richness may produce more useful diagnostic indications of clinical outcomes.

Successful FMT was associated with an increase in diversity and richness generally, and reversals of some of the reported dysbiotic changes in UC. However, the most recent RCT showed an opposite result that the abundance of *Bacteroidetes* in FMT-D responders decreased, which used to be found increasing after FMT, suggesting that UC patients could get clinical remission although the gut microbiota changed in an opposite direction. As Cui et al reported, one patient who did not benefit from the first FMT due to perianal abscess experienced a surgery presented at 7 days after the first FMT, and antibiotics were given before and after surgery. [45] This patient was the only one who did not experienced the increased similarity to donor and increased diversity in their fecal microbiota analyses, indicating that the diversity and composition of fecal microbiota were affected by antibiotic greatly. Therefore, the assumption could be made that some changes of microbiota in UC are induced by inflammation or previous treatment and not the causes of UC universally. Another possibility is that other significant factors of dysbiosis outweighed the composition of bacterial, but no studies have discovered them yet.

Although dysbiosis and its corresponding changes after FMT were similar, the FMT for UC was not as successful as CDI in which FMT led to cure rates of more than 90%. [20] Two different classifications of the gut microbiota, luminal microbiota and mucosal microbiota, [57] may account for this phenomenon. The infection of Clostridium difficile may result in the change of luminal microbiota which could be restored easier by FMT. In contrast, the disruption of mucosa microbiota rather than luminal microbiota is observed in UC patients. Another reason could be that the causative role of dysbiosis in UC and CDI maybe totally different.

### Safety

FMT is generally of safety and tolerance with few serious adverse events. As many patients need to receive more than one FMT therapy, more procedural complications will probably be reported due to the invasion of procedure. Despite rigorous donor selection and screening for infectious agents, known and unknown risks still remain a major problem for widely application of FMT in UC. One of unknown risks is the long-term influence of microbiome on host after FMT. Moreover, short follow-up time increases the risk of underreporting latent adverse events. Therefore, besides the efficacy of FMT, the safety of FMT also needs to be determined especially in long-term follow-up.

### Limitations

The evidence for FMT in this systematic review was mostly depended on two RCTs and 15 cohort studies. The numbers of participants in each study were too small to avoid publication bias and patient selection bias. As an important source of grey literature, conference abstracts are susceptible to report more negative results honestly. So 9 studies we included were available in abstract/letter form. As for the synthesis of cohorts, low or moderate heterogeneity, the results of sensitivity analysis and publish bias all reflected the stability of synthesis. So the synthesis of cohorts was reliable and had reference value for further studies to some extent. There was significant heterogeneity in several aspects. As a novel therapy, FMT has no standard practical guideline for UC, thus we were supposed to take the variations of intervention into account, including the variations in the patient and donor preparation, dosage and frequency, and route of administration. Due to the lack of standardized definitions of clinical outcomes, several indices of disease activity were used including Mayo score, Pediatric Ulcerative Colitis Activity Index (PUCAI), etc., which definitely led to heterogeneity. Mucosal healing offers more objective and accurate evidence to judge the disease activity and therapeutic effects, but only small patients were reported the outcome of mucosal healing.

## Conclusion

Overall, FMT is a promising therapy for UC patients. It could be safe and effective for partial patients. However, the present evidence of FMT for UC is limited and many serious concerns and questions are required to be resolved before it is widely applied. Therefore, more well-designed RCTs and long-term follow-up are necessary to confirm the effects of FMT. Doctors should think over its advantages and disadvantages carefully before recommending and applying it to UC patients.

## Supporting Information

S1 File PRISMA Checklist. (DOC)

S2 File PROSPERO International Prospective Register of Systematic Reviews. (PDF)

S3 File Forest plots and funnel plots of meta-analyses and sensitivity analysis of included studies. (DOCX)

S1 Table Characteristic of case studies. (DOCX)

S2 Table Assessment of Risk of bias in included RCT studies and cohort studies. (DOCX)
