# Supplementary material for: Fecal Microbiota Transplantation for Ulcerative Colitis: A Systematic Review and Meta-Analysis
Source: PLoS One. 2016 Jun 13;11(6):e0157259. doi: 10.1371/journal.pone.0157259 (PMC4905678; doi:10.1371/journal.pone.0157259)
Supplement: S2 File — (PDF) [file pone.0157259.s002.pdf]

# PROSPERO International prospective register of systematic reviews

## Review title and timescale

1 Review title

Give the working title of the review. This must be in English. Ideally it should state succinctly the interventions or exposures being reviewed and the associated health or social problem being addressed in the review.

Fecal microbiota transplantation for ulcerative colitis: a systematic review and/or meta-analysis

2 Original language title

For reviews in languages other than English, this field should be used to enter the title in the language of the review. This will be displayed together with the English language title.

3 Anticipated or actual start date

Give the date when the systematic review commenced, or is expected to commence.

03/08/2015

4 Anticipated completion date

Give the date by which the review is expected to be completed.

01/12/2015

5 Stage of review at time of this submission

Indicate the stage of progress of the review by ticking the relevant boxes. Reviews that have progressed beyond the point of completing data extraction at the time of initial registration are not eligible for inclusion in PROSPERO. This field should be updated when any amendments are made to a published record.

The review has not yet started ×

| Review stage | Started | Completed |
|---|---|---|
| Preliminary searches | Yes | No |
| Piloting of the study selection process | No | No |
| Formal screening of search results against eligibility criteria | No | No |
| Data extraction | No | No |
| Risk of bias (quality) assessment | No | No |
| Data analysis | No | No |

Provide any other relevant information about the stage of the review here.

## Review team details

6 Named contact

The named contact acts as the guarantor for the accuracy of the information presented in the register record.

Pei-zhu Su

7 Named contact email

Enter the electronic mail address of the named contact.

supeizhu1986@163.com

8 Named contact address

Enter the full postal address for the named contact.

Department of Gastroenterology, Zhujiang Hospital, Southern Medical University, 253 Industrial Avenue, Haizhu, Guangzhou, China

9 Named contact phone number

Enter the telephone number for the named contact, including international dialing code.

15521287895

10 Organisational affiliation of the review

Full title of the organisational affiliations for this review, and website address if available. This field may be completed as 'None' if the review is not affiliated to any organisation.

Zhujiang Hospital, Southern Medical University, 253 Industrial Avenue, Haizhu, Guangzhou, 510282, Guangdong, China

Website address:
http://www.zjyy.com.cn/

11 Review team members and their organisational affiliations
Give the title, first name and last name of all members of the team working directly on the review. Give the organisational affiliations of each member of the review team.

| Title | First name | Last name | Affiliation |
|---|---|---|---|
| Mr | Yan-qiang | Shi | Southern Medical University |
| Mrs | Yi-wei | Dong | Southern Medical University |
| Mr | De-cong | Zhu | Southern Medical University |
| Mrs | Wen-hui | Huang | Southern Medical University |
| Dr | Pei-zhu | Su | Zhujiang Hospital, Southern Medical University |

12 Funding sources/sponsors
Give details of the individuals, organizations, groups or other legal entities who take responsibility for initiating, managing, sponsoring and/or financing the review. Any unique identification numbers assigned to the review by the individuals or bodies listed should be included.
None

13 Conflicts of interest
List any conditions that could lead to actual or perceived undue influence on judgements concerning the main topic investigated in the review.
Are there any actual or potential conflicts of interest?
None known

14 Collaborators
Give the name, affiliation and role of any individuals or organisations who are working on the review but who are not listed as review team members.

| Title | First name | Last name | Organisation details |
|---|---|---|---|

## Review methods

15 Review question(s)
State the question(s) to be addressed / review objectives. Please complete a separate box for each question.
What are the efficacy, safety and adverse events rates of fecal microbiota transplantation used for patients with ulcerative colitis?

16 Searches
Give details of the sources to be searched, and any restrictions (e.g. language or publication period). The full search strategy is not required, but may be supplied as a link or attachment.
We will conduct a systematic search to identify all appropriate studies relating to fecal microbiota transplantation used for ulcerative colitis. Ulcerative colitis and fecal microbiota transplantation will be searched using their indexed terms and text words respectively. The search will be conducted using following electronic databases: PubMed, EMBASE, Cochrane Library, Web of Science and ClinialTrials.gov. The specific search strategy will be changed to adapt to the demands of the different electronic databases and will include following words: (fecal OR faecal OR stool OR microbiota OR microflora OR feces OR faeces OR fecal flora OR faecal flora) AND (transplants OR transplant OR transplantation OR transfusion OR implant OR implantation OR instillation OR donor OR enema OR reconstitution OR infusion OR therapy OR bacteriotherapy) AND (ulcerative colitis OR UC OR inflammatory bowel disease OR IBD), some of which were mentioned in previous correlated systematic reviews. There will be no restrictions on study design, date or language. We will also scrutinize the reference lists of the included studies, related reviews and systematic reviews to identify additional studies.

17 URL to search strategy

If you have one, give the link to your search strategy here. Alternatively you can e-mail this to PROSPERO and we will store and link to it.

I give permission for this file to be made publicly available
Yes

18 Condition or domain being studied
Give a short description of the disease, condition or healthcare domain being studied. This could include health and wellbeing outcomes.
Ulcerative colitis is a chronic, relapsing and remitting disease whose precise aetiology is unclear. The treatment of ulcerative colitis is rapidly developing and many novel treatments have proven efficacies. Fecal microbiota transplantation (FMT) is an additional alternative treatment with a promising future as indicated in the research of 2013 which illustrated excellent results limited to patients with Clostridium difficile infections (CDI). It was also used as a therapy in ulcerative colitis with positive outcomes in some studies. However, there is a lack of a thorough analysis of the available evidence which we can use to guide clinical practice and future research.

19 Participants/population
Give summary criteria for the participants or populations being studied by the review. The preferred format includes details of both inclusion and exclusion criteria.
Patients with ulcerative colitis, including adults and children.

20 Intervention(s), exposure(s)
Give full and clear descriptions of the nature of the interventions or the exposures to be reviewed
Ulcerative colitis patients treated with fecal microbiota transplantation.

21 Comparator(s)/control
Where relevant, give details of the alternatives against which the main subject/topic of the review will be compared (e.g. another intervention or a non-exposed control group).
None

22 Types of study to be included initially
Give details of the study designs to be included in the review. If there are no restrictions on the types of study design eligible for inclusion, this should be stated.
We will include randomized controlled trials, case series studies, case reports, cohort studies and case control studies. If studies are only published as abstracts or meeting abstracts but include enough data for our analysis, they will also be included as additional evidence.

23 Context
Give summary details of the setting and other relevant characteristics which help define the inclusion or exclusion criteria.

24 Primary outcome(s)
Give the most important outcomes.
The proportion of patients obtaining clinical remission, clinical response and/or clinical improvement.

Give information on timing and effect measures, as appropriate.

25 Secondary outcomes
List any additional outcomes that will be addressed. If there are no secondary outcomes enter None.
The proportion of patients recording any adverse events. The proportion of patients withdrawing from the fecal microbiota transplantation due to adverse events. The proportion of patients recording serious adverse events, such as death or hospitalization. The proportion of patients recording adverse events potentially due to fecal microbiota transplantation, such as perforation etc. We will analyze the economic data about fecal microbiota transplantation and the timing of outcomes if necessary.

Give information on timing and effect measures, as appropriate.

26 Data extraction, (selection and coding)
Give the procedure for selecting studies for the review and extracting data, including the number of researchers involved and how discrepancies will be resolved. List the data to be extracted.

All records identified by our search strategy will be imported into EndNote and duplicates will be removed. Two of the reviewers will independently determine the eligibility by reading the title and abstract and articles regarded as potentially eligible studies will be obtained as full text. Two reviewers will independently extract useful data from available articles, such as characteristics of patient group, intervention, comparator group, outcome and study design, into a data collection table. If there is any missing data or potential of duplicated data, we will contact the primary authors for the complete data. If there are any disagreements during the process, we will discuss to reach a consensus.

27 Risk of bias (quality) assessment
State whether and how risk of bias will be assessed, how the quality of individual studies will be assessed, and whether and how this will influence the planned synthesis.
The Cochrane Collaboration's tool will be used to assess the level of methodological quality of randomized controlled studies, including bias of selection, performance, detection, attrition, reporting and others. The methodological quality of cohort studies and case control studies will be assessed by the Newcastle-Ottawa Scale, and case series studies will be assessed by the Agency for Healthcare Research and Quality. We will discuss to reach a consensus if disagreements arise.

28 Strategy for data synthesis
Give the planned general approach to be used, for example whether the data to be used will be aggregate or at the level of individual participants, and whether a quantitative or narrative (descriptive) synthesis is planned. Where appropriate a brief outline of analytic approach should be given.
All data from selected publications will be extracted to analyze using a standardized table. If the data achieved is sufficient to conduct a meta-analysis, Review Manager and/or STATA will be used to analyze it using a fixed or random effects model after assessing the heterogeneity. If the data is insufficient to conduct a meta-analysis, we will summarize the overall proportion of patients with events, such as clinical remission etc. and pooled estimation of effect size will be conducted if necessary and appropriate.

29 Analysis of subgroups or subsets
Give any planned exploration of subgroups or subsets within the review. 'None planned' is a valid response if no subgroup analyses are planned.
None planned.

## Review general information

30 Type of review
Select the type of review from the drop down list.
Intervention

31 Language
Select the language(s) in which the review is being written and will be made available, from the drop down list. Use the control key to select more than one language.
English

Will a summary/abstract be made available in English?
Yes

32 Country
Select the country in which the review is being carried out from the drop down list. For multi-national collaborations select all the countries involved. Use the control key to select more than one country.
China

33 Other registration details
Give the name of any organisation where the systematic review title or protocol is registered together with any unique identification number assigned. If extracted data will be stored and made available through a repository such as the Systematic Review Data Repository (SRDR), details and a link should be included here.

34 Reference and/or URL for published protocol
Give the citation for the published protocol, if there is one.
Give the link to the published protocol, if there is one. This may be to an external site or to a protocol deposited with CRD in pdf format.

I give permission for this file to be made publicly available
Yes

35 Dissemination plans
Give brief details of plans for communicating essential messages from the review to the appropriate audiences.
We plan to publish our review in scientific medical journal.

Do you intend to publish the review on completion?
Yes

36 Keywords
Give words or phrases that best describe the review. (One word per box, create a new box for each term)
Fecal microbiota transplantation

Colitis, ulcerative

Humans

37 Details of any existing review of the same topic by the same authors
Give details of earlier versions of the systematic review if an update of an existing review is being registered, including full bibliographic reference if possible.

38 Current review status
Review status should be updated when the review is completed and when it is published.
Ongoing

39 Any additional information
Provide any further information the review team consider relevant to the registration of the review.

40 Details of final report/publication(s)
This field should be left empty until details of the completed review are available.
Give the full citation for the final report or publication of the systematic review.
Give the URL where available.
