# Supplementary material for: Fecal Microbiota Transplantation for Ulcerative Colitis: A Systematic Review and Meta-Analysis
Source: PLoS One. 2016 Jun 13;11(6):e0157259. doi: 10.1371/journal.pone.0157259 (PMC4905678; doi:10.1371/journal.pone.0157259)
Supplement: S3 File — (DOCX) [file pone.0157259.s003.docx]

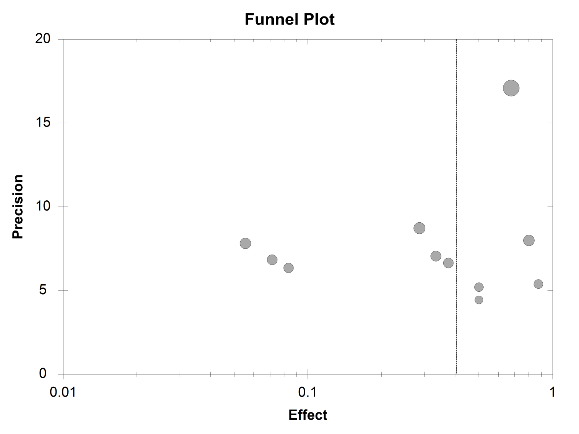


Figure 1-1.

Funnel plot of meta-analysis in clinical remission.


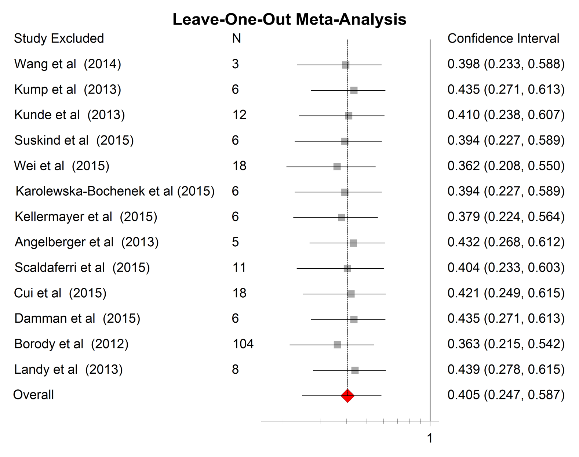


Figure 1-2.

Forest plot of leave-one-out analysis in clinical remission.


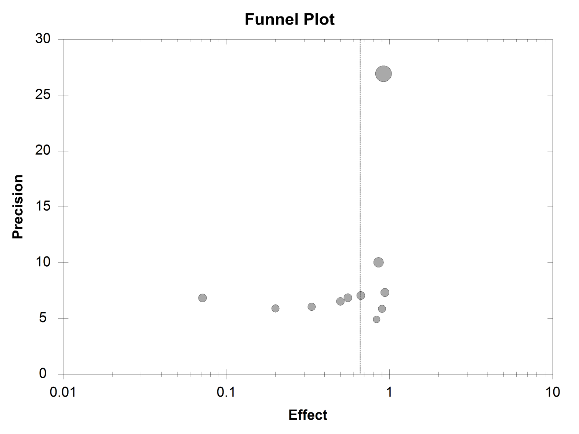


Figure 2-1.

Funnel plot of meta-analysis in clinical response.


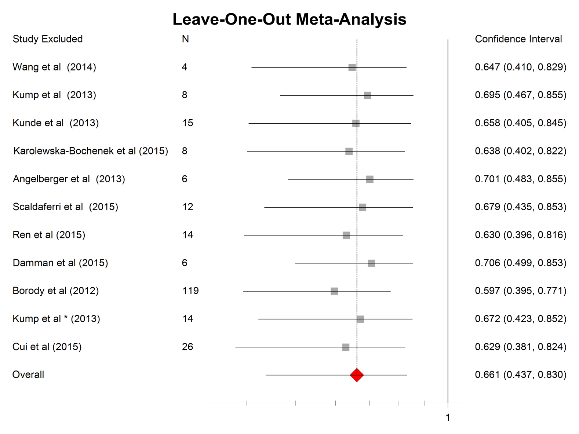


Figure 2-2.

Forest plot of leave-one-out analysis in clinical response.


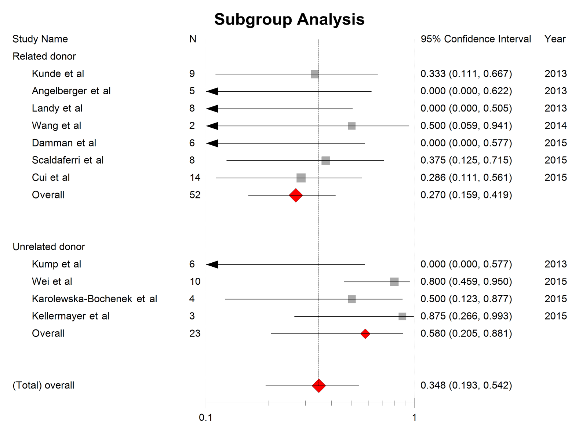


Figure 3-4 a.

Forest plots of subgroup analysis between related donor and unrelated donor in clinical remission with random-effects model.

| Sub-Group | Lower | Estimate | Upper | I^2 | Q | P-Val |
| --- | --- | --- | --- | --- | --- | --- |
| Related donor | 0.159 | 0.270 | 0.419 | 0.000 | 0.827 | 0.364 |
| Unrelated donor | 0.205 | 0.580 | 0.881 | 0.358 | 0.871 | 0.073 |
| Overall | 0.193 | 0.348 | 0.542 | 0.298 | 0.946 | 0.062 |


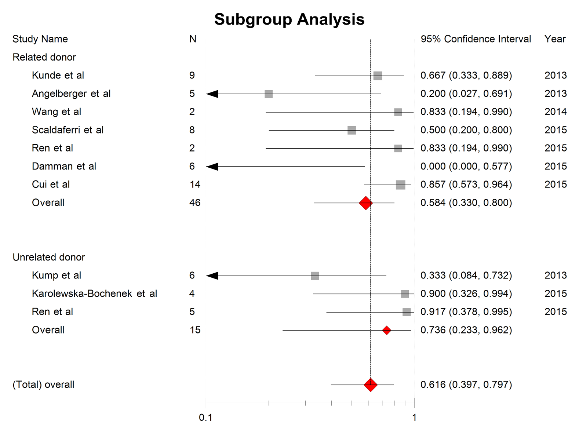


Figure 3-4 b.

Forest plots of subgroup analysis between related donor and unrelated donor in clinical response with random-effects model.

| Sub-Group | Lower | Estimate | Upper | I^2 | Q | P-Val |
| --- | --- | --- | --- | --- | --- | --- |
| Related donor | 0.330 | 0.584 | 0.800 | 0.327 | 0.921 | 0.066 |
| Unrelated donor | 0.233 | 0.736 | 0.962 | 0.370 | 0.829 | 0.081 |
| Overall | 0.397 | 0.616 | 0.797 | 0.313 | 0.943 | 0.054 |


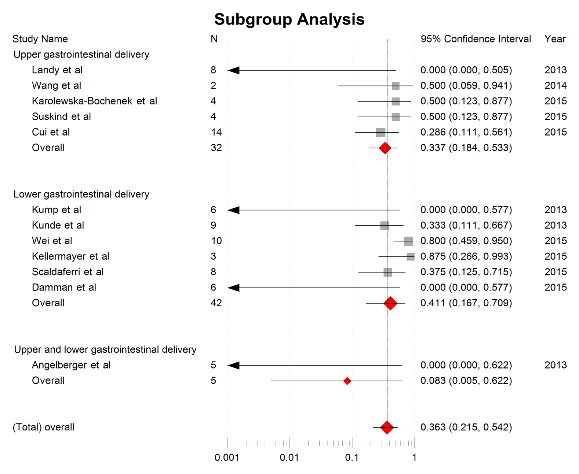


Figure 3-5 a.

Forest plots of subgroup analysis between different routes in clinical remission with random-effects model.

| Sub-Group | Lower | Estimate | Upper | I^2 | Q | P-Val |
| --- | --- | --- | --- | --- | --- | --- |
| Upper gastrointestinal delivery | 0.184 | 0.337 | 0.533 | 0.000 | 0.778 | 0.323 |
| Lower gastrointestinal delivery | 0.167 | 0.411 | 0.709 | 0.367 | 0.922 | 0.035 |
| Upper and lower gastrointestinal delivery | 0.005 | 0.083 | 0.622 | No nunble | 0.000 | 0.024 |
| Overall | 0.215 | 0.363 | 0.542 | 0.274 | 0.946 | 0.082 |


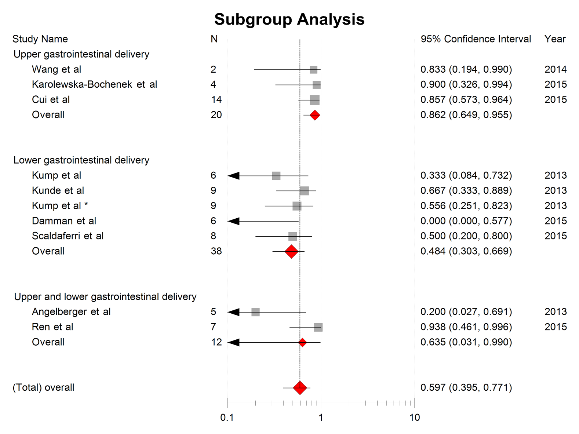


Figure 3-5 b.

Forest plots of subgroup analysis between different routes in clinical response with random-effects model.

| Sub-Group | Lower | Estimate | Upper | I^2 | Q | P-Val |
| --- | --- | --- | --- | --- | --- | --- |
| Upper gastrointestinal delivery | 0.649 | 0.862 | 0.955 | 0.000 | 0.077 | 0.490 |
| Lower gastrointestinal delivery | 0.303 | 0.484 | 0.669 | 0.138 | 0.826 | 0.238 |
| Upper and lower gastrointestinal delivery | 0.031 | 0.635 | 0.990 | 0.444 | 0.832 | 0.025 |
| Overall | 0.395 | 0.597 | 0.771 | 0.315 | 0.943 | 0.051 |


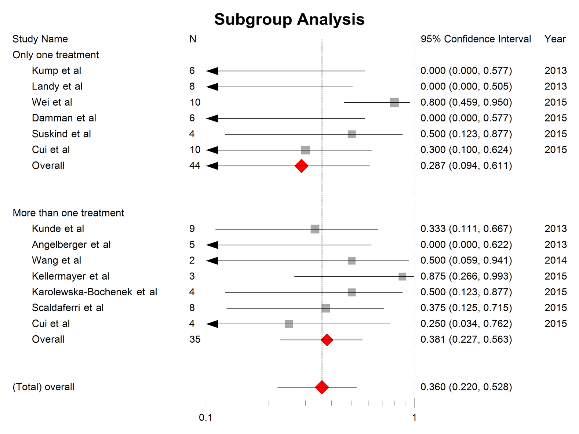


Figure 3-6 a.

Forest plots of subgroup analysis between only one treatment and more than one treatment in clinical remission with random-effects model.

| Sub-Group | Lower | Estimate | Upper | I^2 | Q | P-Val |
| --- | --- | --- | --- | --- | --- | --- |
| Only one treatment | 0.094 | 0.287 | 0.611 | 0.378 | 0.927 | 0.026 |
| More than one treatment | 0.227 | 0.381 | 0.563 | 0.000 | 0.833 | 0.353 |
| Overall | 0.220 | 0.360 | 0.528 | 0.244 | 0.947 | 0.111 |


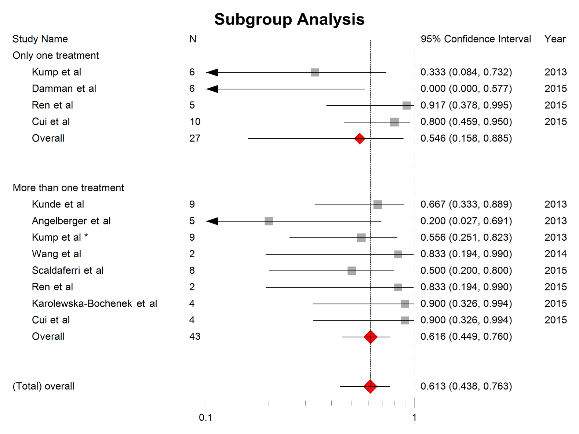


Figure 3-6 b.

Forest plots of subgroup analysis between only one treatment and more than one treatment in clinical response with random-effects model.

| Sub-Group | Lower | Estimate | Upper | I^2 | Q | P-Val |
| --- | --- | --- | --- | --- | --- | --- |
| Only one treatment | 0.158 | 0.546 | 0.885 | 0.400 | 0.900 | 0.028 |
| More than one treatment | 0.449 | 0.616 | 0.760 | 0.027 | 0.878 | 0.290 |
| Overall | 0.438 | 0.613 | 0.763 | 0.245 | 0.942 | 0.115 |
