# Supplementary material for: Fecal Microbiota Transplantation for Ulcerative Colitis: A Systematic Review and Meta-Analysis
Source: PLoS One. 2016 Jun 13;11(6):e0157259. doi: 10.1371/journal.pone.0157259 (PMC4905678; doi:10.1371/journal.pone.0157259)
Supplement: S1 Table — (DOCX) [file pone.0157259.s004.docx]

Table. Characteristic of case studies.

| Characteristic of studies | | | Characteristics of patients | | |
| --- | --- | --- | --- | --- | --- |
| Study (Year, Reference) | Country | Type | Number (M/F) | Age | Severity (duration) |
| Hohmann *et al*.^52^(2014) | America | Journal article | 1(1/0) | 37y | Severe (7y) |
| Bennet *et al.*^47^(1989) | America | Letter | 1(1/0) | NR | Severe |
| Borody *et al.*^49^(2003) | Australia | Journal article | 6(3/3) | 35.8y | Severe (12y) |
| Borody *et al.*^48^(1989) | Australia | Letter | 1(1/0) | 45y | Active (18m) |
| Borody *et al.*^50^(2011) | Australia | Abstract | 1(0/1) | 39y | Chronic relapsing with ITP |
| Ni *et al.*^53^(2015) | China | Journal article | 1(1/0) | 24y | Severe (6y) |
| Liu *et al.*^51^(2013) | China | Journal article | 1(0/1) | 28y | Severe (12y) |
| Vandenplas *et al.*^54^(2014) | Netherlands | Journal article | 1(0/1) | 18 m | Moderate-severe |

PEC, percutaneous endoscopic cecostomy; ITP, idiopathic thrombocytopenic purpura, NR, not reported.

Table. Characteristic of case studies. (Continued)

| Characteristic of studies | Characteristics of intervention | | | | |
| --- | --- | --- | --- | --- | --- |
| Study (Year, Reference) | Patient preparation | Donor | Stool processing | The route of delivery | Number of treatment |
| Hohmann *et al*.^52^(2014) | NR | Wife and child | NR | NR | 4 treatments |
| Bennet *et al.*^47^(1989) | "Sterilised bowel" | NR | Large volume | Enema | ﹥1 |
| Borody *et al.*^49^(2003) | Antibiotics/ oral PEG 3L | 3 brothers; 1 partner; 1 unrelated; 1 brother in law. | 200-300g/200-300ml saline | Enema | Daily×5 |
| Borody *et al.*^48^(1989) | NR | NR | NR | NR | NR |
| Borody *et al.*^50^ (2011) | NR | NR | NR | NR | NR |
| Ni *et al.*^53^(2015) | Mesalazine | Father | 100g/250 ml saline | PEC | 40 treatments |
| Liu *et al.*^51^(2013) | NR | Husband | NR | Enema | Every 3 days ×3 |
| Vandenplas *et al.*^54^(2014) | NR | Niece and older brother | 100g/100 ml saline | Colonoscopy + nasoduodenal tube | 7 treatments |

Table. Characteristic of case studies. (Continued)

| Characteristic of studies | Characteristics of outcomes | | |
| --- | --- | --- | --- |
| Study (Year, Reference) | Clinical outcome | Adverse | Fllow-up |
| Hohmann *et al*.^52^(2014) | Cytomegalovirus colitis (home FMT). | Symptoms related to cytomegalovirus infection. | Several weeks |
| Bennet *et al.*^47^(1989) | Patient achieved asymptom for first time in 11 years without any medication (no bloody diarrhea, cramping, tenesmus, skin lesions, and arthritis); Biopsy specimens revealed chronic inflammation. | NR | 6 months |
| Borody *et al.*^49^(2003) | All patients achieved asymptoms and all UC medications were ceased; Colonoscopy and histopathology were normal at long-term follow-up | NR | 1-13 years |
| Borody *et al.*^48^(1989) | All treatments were ceased; No diarrhea and no any medication; colonoscopic examination and mucosal biopsies were normal. | NR | 3 months |
| Borody *et al.*^50^(2011) | The platelet count normalized and the immune-mediated ITP successfully reversed. The symptoms of UC was markly reduced. | NR | ＞10 years |
| Ni *et al.*^53^(2015) | Symptoms were resolved and endoscopic examination showed mucosa normal. | None | 2 months |
| Liu *et al.*^51^(2013) | Symptoms were resolved and Mayo score was 3. | NR | 3 weeks |
| Vandenplas *et al.*^54^(2014) | Symptoms were resolved. | Profuse sweating, vomiting, tachycardia, paleness and transient fever. | 6 months |
