# Supplementary material for: Fecal Microbiota Transplantation for Ulcerative Colitis: A Systematic Review and Meta-Analysis
Source: PLoS One. 2016 Jun 13;11(6):e0157259. doi: 10.1371/journal.pone.0157259 (PMC4905678; doi:10.1371/journal.pone.0157259)
Supplement: S2 Table — (DOCX) [file pone.0157259.s005.docx]

Table.1 Assessment of Risk of bias in included RCT studies.

| Study | Adequate sequence generation | Allocation concealment | Blinding of participants and personnel | Blinding of outcome assessment | Incomplete outcome data | selective reporting | Other bias |
| --- | --- | --- | --- | --- | --- | --- | --- |
| Moayyedi et al.^22^ | Low | Low | Low | Low | Unclear | Low | Unclear |
| Rossen et al.^23^ | Unclear | Unclear | Low | Low | Low | Low | Unclear |

Table.2 Assessment of Risk of bias in included cohort studies.

| Quality assessment scale | | Kump et al. ^35^ (2013) | Kunde et al. ^36^ (2013) | Suskind et al. ^43^ (2015) | Wei  et al.^44^  (2015) | Karolewska-Bochenek et al.^46^  (2015) | Kellermayer et al.^41^  (2015) | Angelberger et al.^34^  (2013) |
| --- | --- | --- | --- | --- | --- | --- | --- | --- |
| Selection | Representativeness of the exposed cohort. | - | * | - | * | * | - | - |
|  | Ascertainment of exposure. | * | * | * | * | * | * | * |
|  | Demonstration that outcome of interest was not present at start of study. | * | - | - | * | - | - | * |
| Outcome | Assessment of outcome | * | * | * | * | * | * | * |
|  | Was follow-up long enough for outcomes to occur. | * | - | * | - | - | * | * |
|  | Adequacy of follow up of cohorts | * | * | * | * | * | * | * |
| Total |  | 5 | 4 | 4 | 5 | 4 | 4 | 5 |

Table2. Assessment of Risk of bias in included cohort studies. (Continued)

| Quality assessment scale | | Scaldaferri et al.^32^  (2015) | Ren  et al.^42^  (2015) | Cui et al.^45^ (2015) | Damman et al.^40^  (2015) | Borody et al.^33^  (2012) | Kump  et al.^38^  (2013) | Wang  et al.^39^  (2014) | Landy  et al.^37^  (2013) |
| --- | --- | --- | --- | --- | --- | --- | --- | --- | --- |
| Selection | Representativeness of the exposed cohort. | * | - | * | * | * | - | - | - |
|  | Ascertainment of exposure. | * | * | * | * | * | * | * | * |
|  | Demonstration that outcome of interest was not present at start of study. | - | - | - | - | - | - | - | - |
| Outcome | Assessment of outcome | * | * | * | * | * | * | * | * |
|  | Was follow-up long enough for outcomes to occur. | * | * | * | * | - | * | * | - |
|  | Adequacy of follow up of cohorts | * | - | * | * | * | * | * | * |
| Total |  | 5 | 3 | 5 | 5 | 4 | 4 | 4 | 3 |
